# Arabidopsis Chloroplast protein for Growth and Fertility1 (CGF1) and CGF2 are essential for chloroplast development and female gametogenesis

**DOI:** 10.1186/s12870-020-02393-5

**Published:** 2020-04-19

**Authors:** Rui-Min Zhu, Sen Chai, Zhuang-Zhuang Zhang, Chang-Le Ma, Yan Zhang, Sha Li

**Affiliations:** 1 State Key laboratory of Crop Biology, College of Life Sciences, Shandong Agricultural University, Tai’an, 271018 China; 2 Shandong Provincial Key Laboratory of Plant Stress, College of Life Science, Shandong Normal University, Jinan, 250014 China

**Keywords:** Chloroplast, Dwarfism, Ovule development, Plastid, Embryogenesis

## Abstract

**Background:**

Chloroplasts are essential organelles of plant cells for not only being the energy factory but also making plant cells adaptable to different environmental stimuli. The nuclear genome encodes most of the chloroplast proteins, among which a large percentage of membrane proteins have yet to be functionally characterized.

**Results:**

We report here functional characterization of two nuclear-encoded chloroplast proteins, Chloroplast protein for Growth and Fertility (CGF1) and CGF2. *CGF1* and *CGF2* are expressed in diverse tissues and developmental stages. Proteins they encode are associated with chloroplasts through a N-terminal chloroplast-targeting signal in green tissues but also located at plastids in roots and seeds. Mutants of *CGF1* and *CGF2* generated by CRISPR/Cas9 exhibited vegetative defects, including reduced leaf size, dwarfism, and abnormal cell death. *CGF1* and *CGF2* redundantly mediate female gametogenesis, likely by securing local energy supply. Indeed, mutations of both genes impaired chloroplast integrity whereas exogenous sucrose rescued the growth defects of the *CGF* double mutant.

**Conclusion:**

This study reports that two nuclear-encoded chloroplast proteins, Chloroplast protein for Growth and Fertility (CGF1) and CGF2, play important roles in vegetative growth, in female gametogenesis, and in embryogenesis likely by mediating chloroplast integrity and development.

## Background

Chloroplasts are light-harvesting organelles essential for plant survival. Chloroplasts contains three sub-compartments separated by the outer envelope, inner envelope, and thylakoid membranes [1]. Although the main function of chloroplasts is oxygenic photobiosynthesis, it also produces various compounds, such as phytohormones, fatty acids, vitamins, as well as secondary metabolites that are indispensable for plant physiology and development [2]. Chloroplasts, and plastids as in non-greening cells, also respond to environmental stimuli, such as gravity and stresses, to make plants more adaptable [3–6].

Chloroplasts are derived from ancestral cyanobacteria. They seamlessly integrate into and coevolve with their host cells to become an integral part of the modern plant cell [7–10]. A large number of genes, mostly involved in development, metabolism, and environmental responses, have been transferred from the engulfed cyanobacteria to the nuclear genome of their host cells for mutual benefits [8]. Chloroplast genomes only encode about 80 to 100 proteins, while between 2500 and 3500 nuclear-encoded proteins are predicted to be targeted to chloroplasts [1].

Chloroplast proteins encoded by the nuclear genome often play critical roles in maintaining chloroplast development and activity. Functional loss of *Thylakoid Formation1* (*THF1*) resulted in slow and uneven chloroplast development due to defective etioplast development in the dark [11]. Vesicle-Inducing Protein in Plastids 1 (VIPP1) is critical for the maintenance of chloroplast envelope and thus chloroplast development [12]. Functional loss of Stromal Processing Peptidase (SPP) compromised chloroplast biogenesis and resulted in embryo lethality [13] whereas two other proteases, VAR2 (yellow variegated 2) and EVR3 (enhancer of variegation 3) also regulate chloroplast development in Arabidopsis [14]. Chloroplast-localized Pentatricopeptide Repeat 287 (PPR287) is essential for chloroplast biogenesis and function, whose downregulation resulted in yellowish leaves, shorter roots and dwarfism, and reduced seed yield [15].

Proteomic studies reveal over 100 membrane proteins at chloroplast envelop in Arabidopsis, among which one third has no known function [16]. Two of those proteins, hereafter named Chloroplast protein for Growth and Fertility (CGF1) and CGF2, are highly homologous with each other but not with any other proteins encoded in the Arabidopsis genome. Here, we report that Arabidopsis CGF1 and CGF2 are important for plant development by mediating chloroplast integrity and possibly development. We demonstrate that *CGF1* and *CGF2* are constitutively expressed. Proteins they encode are associated with chloroplasts through a N-terminal chloroplast-targeting signal. Mutants of *CGF1* and *CGF2* generated by CRISPR/Cas9 exhibited vegetative and reproductive defects, indicative of chloroplast malfunction. Indeed, functional loss of both genes impaired the integrity of chloroplasts. Results presented will facilitate a better understanding of the light-harvesting organelle.

## Results

### *CGF1* and *CGF2* are expressed in diverse tissues and developmental stages

**Fig. 1:**
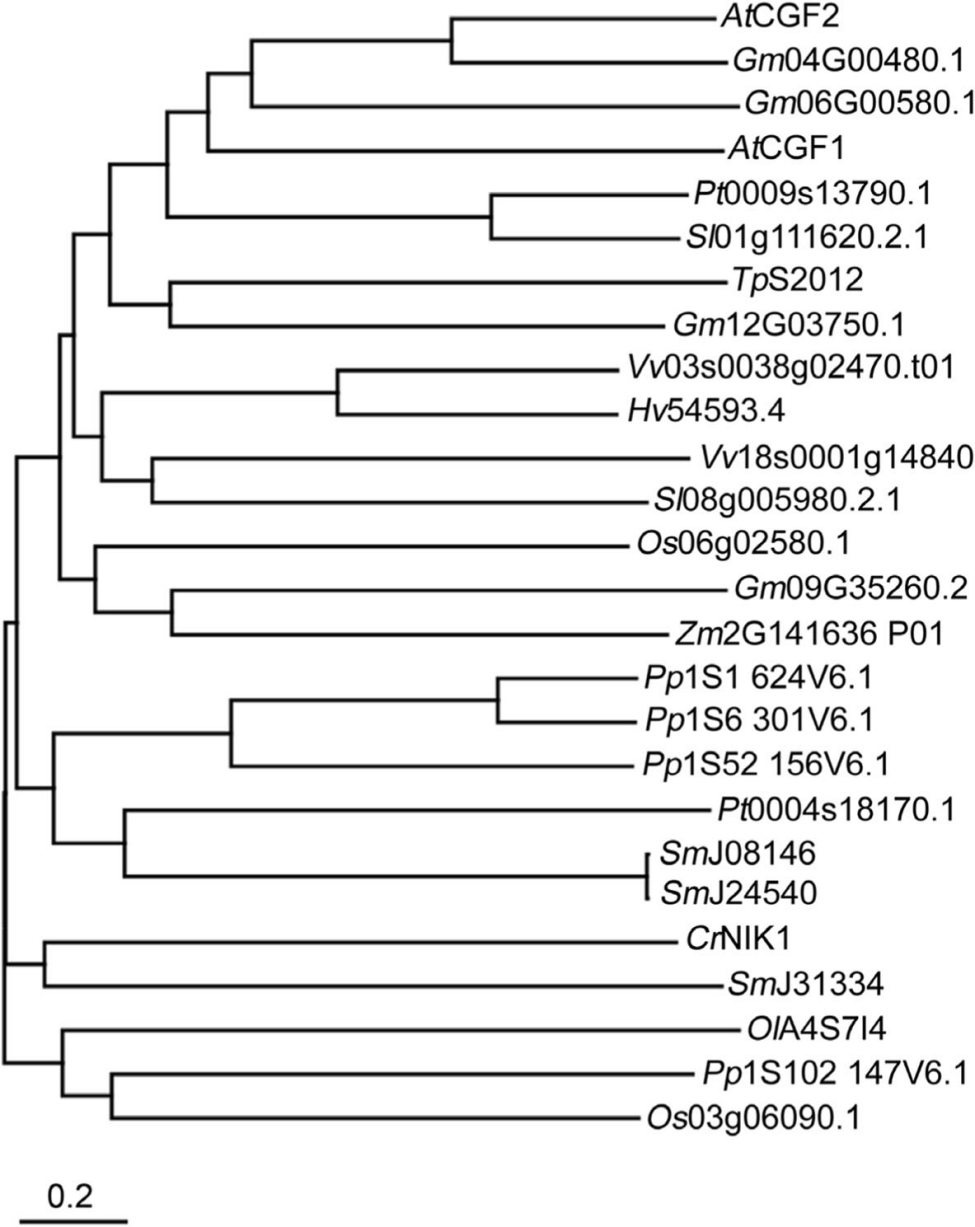
Phylogenetic analysis of CGFs in different species. Protein sequence analysis used MEGA7.0 software. Arabidopsis protein sequences were obtained from TAIR, whereas proteins from other species were obtained from the National Center for Biotechnology Information. Species prefixes are as follows: *Vv*, *Vitis vinifera*; *Cr*, *Chlamydomonas reinhardtii*; *Pp*, *Physcomitrella patens*; *Tp*, *Thalassiosira pseudonana*; *Pt*, *Populus trichocarpa*; *At*, *Arabidopsis thaliana*; *Gm*, *Glycine max*; *Os*, *Oryza sativa*; *Sl*, *Solanum lycopersicum*; *Ol*, *Ostreococcus lucimarinus CCE9901*; *Sm*, *Selaginella moellendorffii*; *Zm*, *Zea mays*; *Hv, Hordeum vulgare*. Scale bar indicates the average number of amino acid substitutions per site

**Fig. 2:**
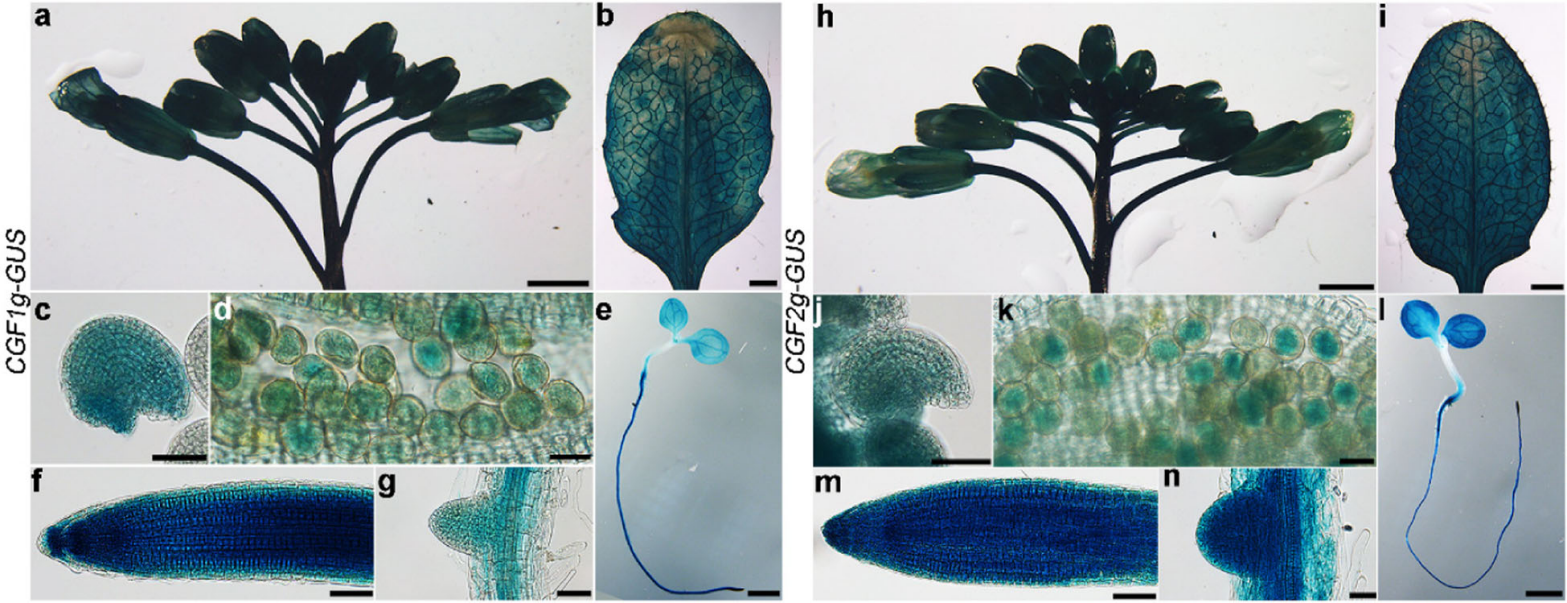
*CGF1* and *CGF2* are expressed in diverse tissues and developmental stages. **a-n** Histochemical GUS staining of an inflorescence **a, h**, a leaf **b, i**, ovules **c, j**, mature pollen grains **d, k**, a seedling **e, l**, a primary root **f, m**, a lateral root **g, n** from CGF1g-GUS **a-g** or CGF2g-GUS **h-n** transgenic plants. Bars = 1 mm for **a, b, e, h, i, l**; 50 μm for **c, f, j, m**; 20 μm for **d, g, k, n**

CGF1 and CGF2 were identified in proteomic studies of chloroplasts or chloroplast envelope proteins in *Arabidopsis* [16]. These two proteins have multiple transmembrane (TM) domains (Figure S1), potentially as metal-transporters based on annotations (www.Arabidopsis.org). Although predicted to have different number of TM domains (Figure S1), CGF1 and CGF2 share 67.4% similarity in amino acid sequences (Figure S1), implying functional redundancy. Sequence-based searches showed that there are CGF homologs from *Chlamydomonas* to monocots and dicots but not in budding yeast or cynobacteria (Fig. 1). To explore the physiological function of *CGF1* and *CGF2*, we first examined the expression patterns of *CGF1* and *CGF2* by quantitative real-time PCR (qPCR) and by genomic-GUS reporter analysis. *CGF1* and *CGF2* were expressed in diverse tissues and developmental stages based on qPCRs (Figure S2). Histochemical GUS staining of the transgenic plants expressing their genomic-GUS fusions, CGF1g-GUS or CGF2g-GUS, confirmed the wide-spread expression of both genes (Fig. 2). Strong GUS signals were detected in leaves and inflorescence including mature pollen and ovules (Fig. 2). In addition, underground tissues such as roots were also GUS-positive (Fig. 2), suggesting that CGFs play roles in diverse tissues and developmental stages.

### CGF1 and CGF2 are associated with chloroplasts and plastids

**Fig. 3:**
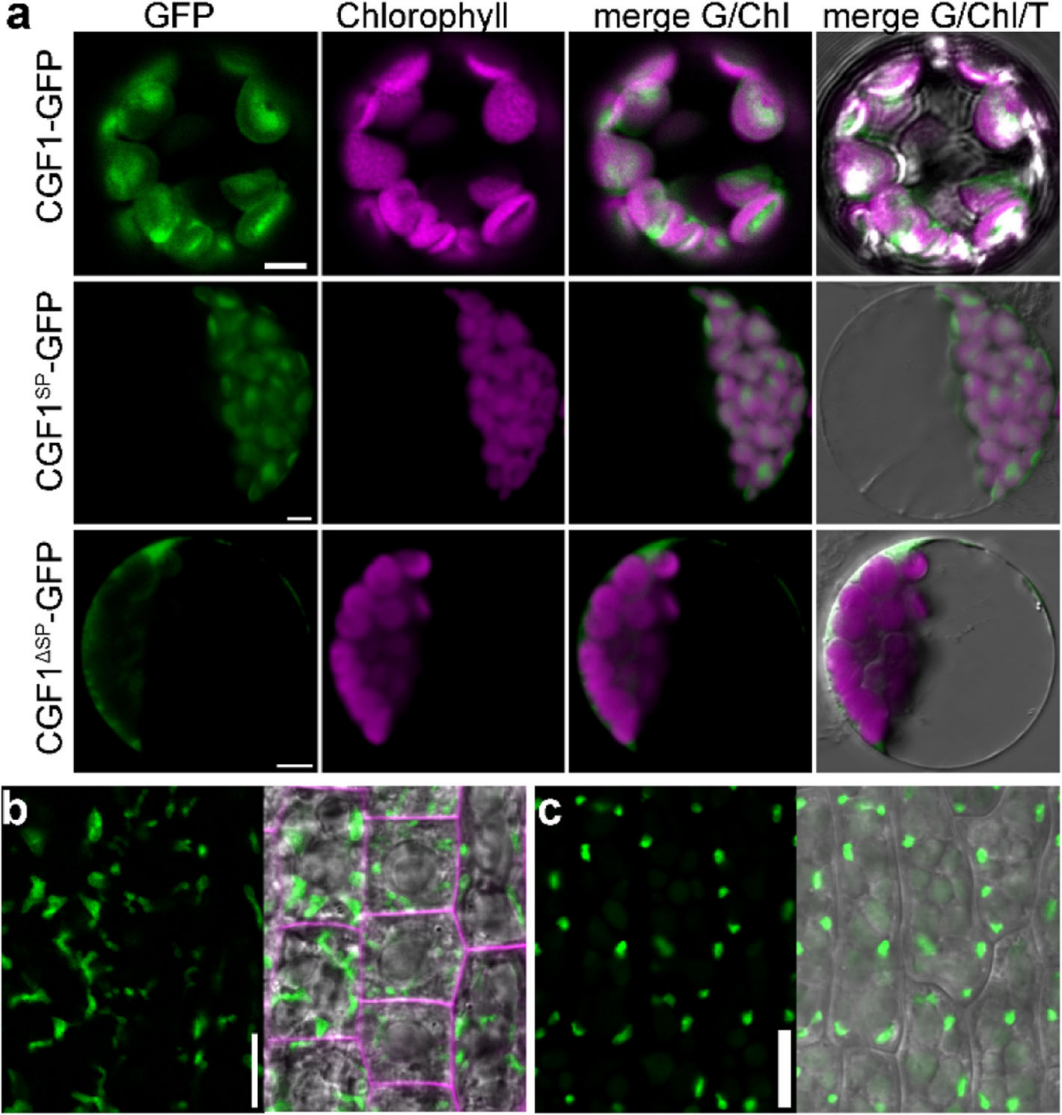
CGF1 targets to chloroplasts through its N-terminal sequences. **a** CLSM of protoplasts from *Pro*_*UBQ10*_:CGF1-GFP, *Pro*_*35S*_:CGF1^SP^-GFP*, or Pro*_*35S*_:CGF1^ΔSP^-GFP transgenic plants. From left to right: the GFP channel, autofluorescence channel (chlorophyll), merge of the GFP and autofluorescence (Chl) channels, merge of the GFP, autofluorescence, and transmission channels. **b** CLSM of root epidermal cells from *Pro*_*UBQ10*_:CGF1-GFP. The right image is the merge of the GFP, RFP (FM4–64), and transmission channels. **c** CLSM of pavement cells from embryonic cotyledons of the *Pro*_*UBQ10*_:CGF1-GFP transgenic plants. The right image is the merge of the GFP and transmission channels. Bars = 5 μm for **a**; 10 μm for **b-c**

CGF1 and CGF2 both contain several TM domains and a chloroplast transit peptide sequence (cTP) in its N-terminus based on analyses using the online tools HMMTOP (http://www.enzim.hu/hmmtop/html/submit.html) and TargetP1.1 (http://www.cbs.dtu.dk/services/TargetP-1.1/index.php). To determine the subcellular localization of CGF1 and CGF2, we generated *Pro*_*UBQ10*_:CGF1-GFP and *Pro*_*UBQ10*_:CGF2-GFP transgenic plants. Confocal laser scanning microscopic (CLSM) analysis of leaf protoplasts from these transgenic plants showed that both proteins are associated with chloroplasts (Fig. 3a and Figure S3). To verify the predicted chloroplast transit peptide sequences in their N-termini, we expressed GFP-fused CGF1 and CGF2 as truncations, i.e. CGF1^SP^ (containing 1–79 aa as its cTP), CGF2^SP^ (containing 1–78 aa as its cTP), CGF1^ΔSP^ (containing 80–365 aa), and CGF2^ΔSP^ (containing 79–372 aa). CLSM analysis of protoplasts expressing these truncated proteins showed that truncations without the cTP sequences were targeted to the cytoplasm or the plasma membrane whereas cTP sequences were able to direct GFP to chloroplasts (Fig. 3a and Figure S3). These results suggested that the predicted cTP sequences are necessary and sufficient for the chloroplast targeting of both CGF1 and CGF2.

Because *CGFs* are also expressed in non-greening tissues/cells (Fig. 2) where plastids instead of chloroplasts are present. We thus examined the subcellular targeting of CGFs in root epidermal cells and in maturing embryos. In root epidermal cells as well as in maturing embryos, both CGF1 and CGF2 are targeted to plastids (Fig. 3b-c and Figure S3).

### *CGF1* and *CGF2* are essential for viability

**Fig. 4:**
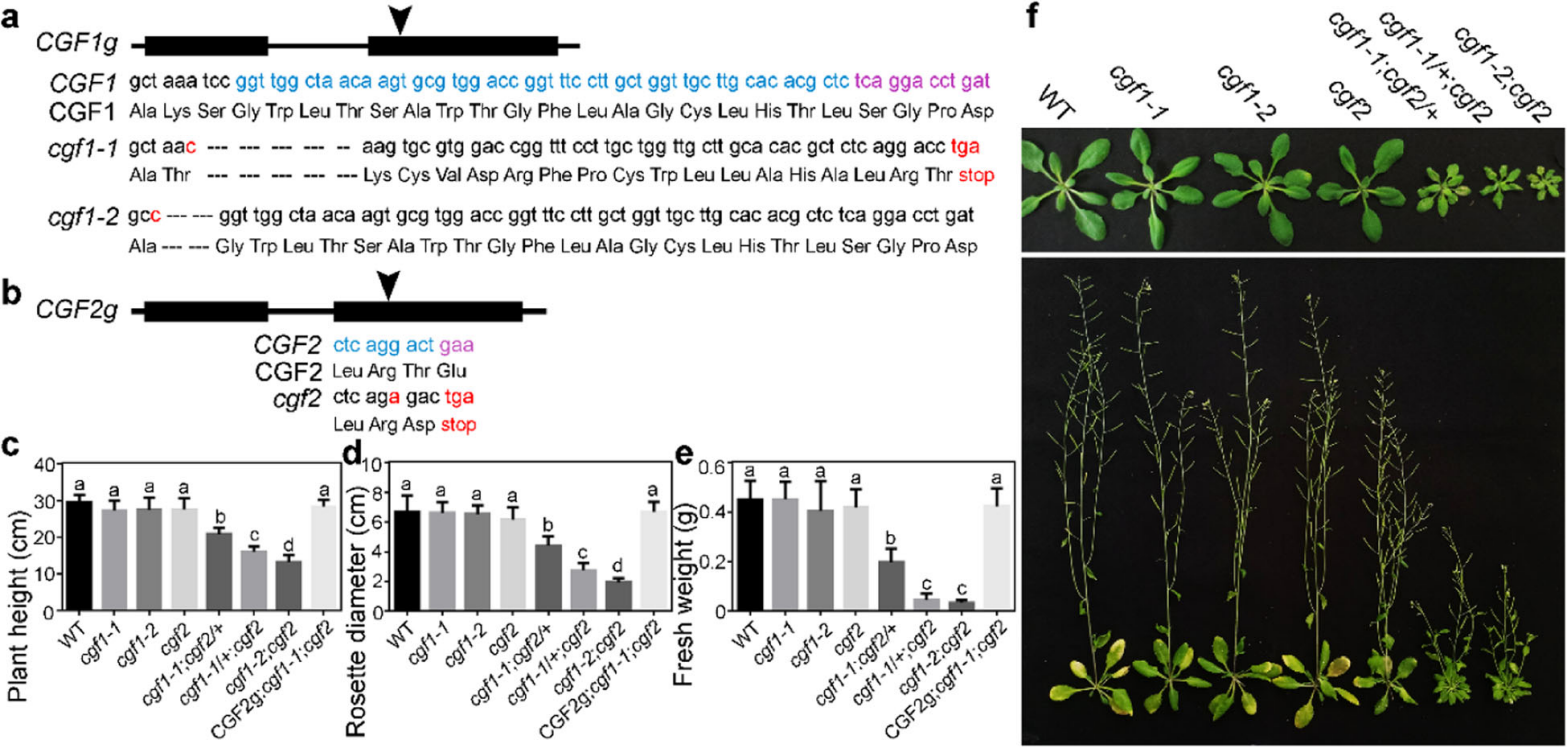
*CGF1* and *CGF2* are essential for viability. **a-b** Schematic illustration of the *CGF1***a** or *CGF2* genomic locus **b** and the generation of CRISPR/Cas9 mutants. Arrowheads point at the genomic locus where Cas9 is targeted. **c-e** Plant height at 7 WAG **c**, rosette diameters at 4 WAG **d**, or fresh weight at 4 WAG **e** of wild type, *cgf1–1*, *cgf1–2*, *cgf2*, *cfg1–1*/+;*cgf2*, *cgf1–1*;*cgf2*/+, *cgf1–2;cgf2*, or CGF2g;*cgf1–1*;*cgf2*. Results are means ± SD (*n* = 15). Different letters indicate significant different groups (OneWay ANOVA, Tukey’s multiple comparisons test, *P* < 0.05). (f) Representative vegetative (top panel) or reproductive growth (bottom panel) of designated genotypes

Because no T-DNA lines of *CGF1* and *CGF2* from stock centers were verified to have insertion in their respective genomic locus, we generated mutants, *cgf1–1*, *cgf1–2*, and *cgf2*, by CRISPR/Cas9 (Fig. 4a, b). Specifically, a 14 bp deletion in the coding sequence of *CGF1* resulted in a pre-stop codon after 942 bp in *cgf1–1* while a 6 bp deletion in *cgf1–2* potentially resulted in a deletion of two amino acids in CGF1 (Fig. 4a). The *cgf2* mutant was generated by one base-pair insertion, which resulted a pre-stop codon (Fig. 4b). All three single mutants were comparable to wild type during vegetative and reproductive growth (Fig. 4c-f), likely due to redundancy. To test this possibility, we generated double mutants by crosses. No homozygous *cgf1–1;cgf2* plants could be obtained despite that more than 600 plants at F2 generation were sequenced. Segregation ratio of the self-fertilized *cgf1–1*/+*;cgf2*/+ indicated that the double mutant results in embryo or seedling lethality (Table S1). By contrast, the double mutant *cgf1–2;cgf2* was obtained (Fig. 4c-f) likely because that *cgf1–2* is a weak allele. This is consistent with the fact that CGF1 potentially encoded in *cgf1–2* only lacks two amino acids whereas that in *cgf1–1* is truncated (Fig. 4a-b).

Analysis of plant growth showed that *cgf1–2;cgf2* was significantly reduced in plant height (Fig. 4c, f), rosette diameter (Fig. 4d, f), and fresh weight (Fig. 4e). Interestingly, two heterozygous double mutants *cgf1–1;cgf2/+* and *cgf1–1/+;cgf2* were also defective in growth (Fig. 4c-f), similar to that of *cgf1–2;cgf2*, indicating haploinsufficiency. Introducing a genomic *CGF2* fragment complemented the growth defects of *cgf1–2;cgf2* (Fig. 4c-f) whereas downregulating *CGF1* in *cgf2* mimicked the growth defects of *cgf1–2;cgf2* (Figure S4), both supporting a key role of *CGFs* in plant growth.

### *CGFs* play roles in female gametogenesis and embryogenesis

**Fig. 5:**
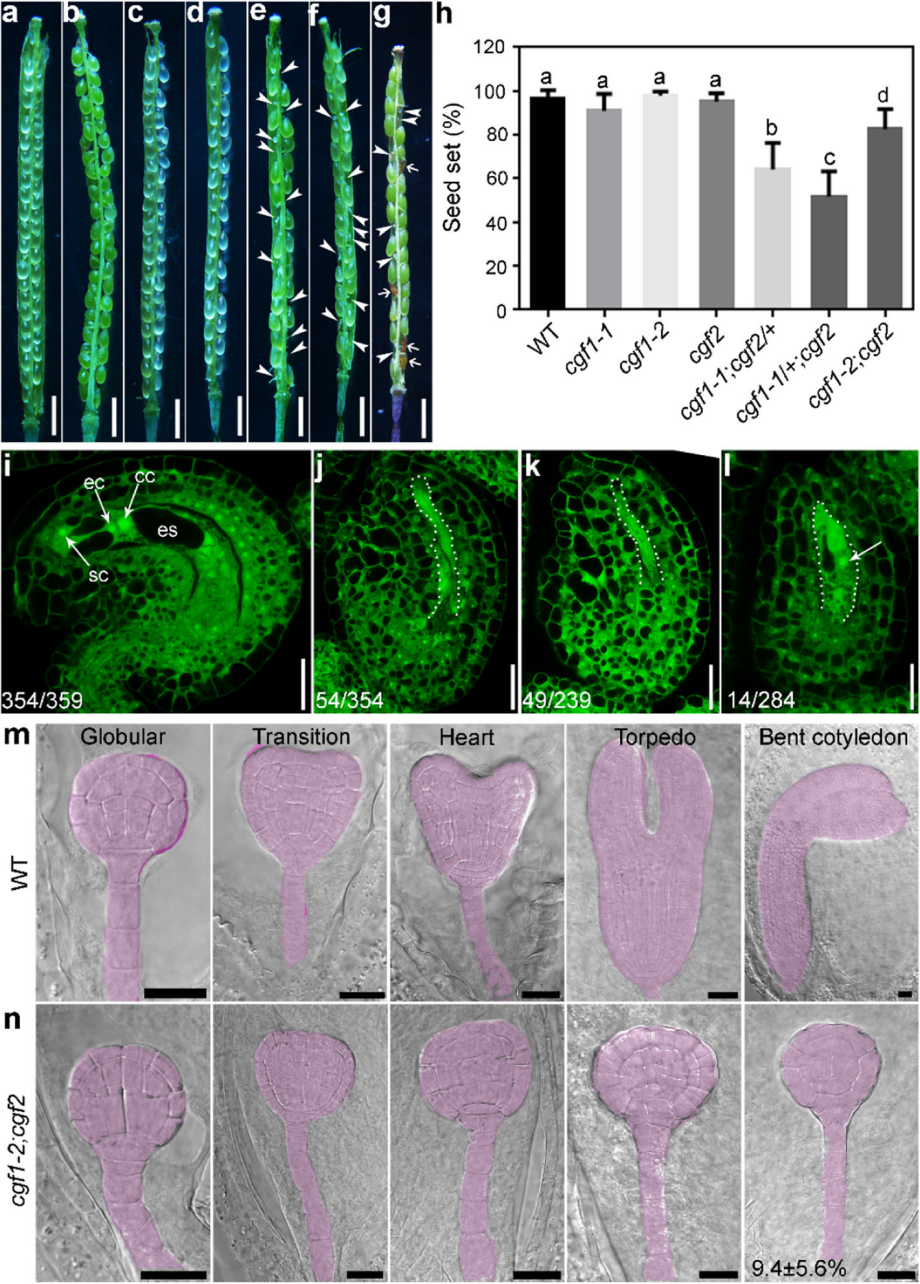
Reduced fertility of the *cgf1;cgf2* double mutants is due to compromised female gametogenesis or embryogenesis. **a-g** A representative silique from wild type **a**, *cgf1–1***b**, *cgf1–2***c**, *cgf2***d**, *cgf1–1*;*cgf2*/+ **e**, *cfg1–1*/+;*cgf2***f**,or *cgf1–2;cgf2***g**. Arrowheads point at unfertilized ovules; arrows point at aborted seeds. **h** Seed set. Results are means ± SD (*n* > 10). Different letters indicate significantly different groups (OneWay ANOVA, Tukey’s multiple comparisons test, *P* < 0.05). **i-l** CLSM of a mature ovule from wild type **i**, *cgf1–1*;*cgf2*/+ **j**, *cfg1–1*/+;*cgf2***k**,or *cgf1–2;cgf2***l**. cc, central cell; ec, egg cell; es, embryo sac; sc, synergid cell. Dotted lines in **j-l** illustrate defective embryo sacs. The arrow in **l** points at a single nucleus. Numbers at the bottom indicate displayed ovules/total ovules examined. **m-n** Differential interference contrast (DIC) imaging of developing embryos in wild type **m** or in *cgf1–2;cgf2***n**. Developing embryos are highlighted with lilac. Defective embryogenesis in *cgf1–2;cgf2* is 9.4 ± 5.6% (*n* > 15). Bars = 1 mm for **a-g**; 20 μm for **i-n**

By silique analysis, we observed the presence of wrinkled, white ovules in developing siliques of *cgf1–1*;*cgf2*/+*, cgf1–1*/+;*cgf2*, and *cgf1–2*;*cgf2* (Fig. 5a-h). Wrinkled, white ovules are usually consequences of failed fertilization [17, 18]. To determine whether this was the case, we examined mature ovules by confocal laser scanning microscopy (CLSM). Optical sections of wild-type ovules showed a well-patterned embryo sac with a central vacuole, central cell nucleus, egg cell nucleus, and synergid nuclei surrounded by integument cells (Fig. 5i). By contrast, a portion of ovules from *cgf1–1*;*cgf2*/+*, cgf1–1*/+;*cgf2*, and *cgf1–2*;*cgf2* did not contain embryo sac structure although integuments seemed normal (Fig. 5j-l). The defective embryo sac structure in *cgf1–1*;*cgf2*/+*, cgf1–1*/+;*cgf2*, and *cgf1–2*;*cgf2* would have caused the reduced fertility. Indeed, pollen from *cgf1–1*;*cgf2*/+*, cgf1–1*/+;*cgf2*, and *cgf1–2*;*cgf2* showed no difference from that of wild type (Figure S5), supporting that the reduced fertility was from the female rather than male side.

In addition to defective ovule development, the homozygous double mutant *cgf1–2*;*cgf2* contained some brownish seeds in its developing siliques (Fig. 5g). To determine the cause of seed abortion in *cgf1–2*;*cgf2*, we examined developing embryos during time course by whole-mount clearing assays. Embryos in wild type develop from early globular stage to the bend cotyledon stage from 3 days after fertilization (DAF) to 10 DAF (Fig. 5m), as reported [19]. Embryos in the siliques of *cgf1–2*;*cgf2* were comparable to those of wild type before the globular stage (Fig. 5n). However, a few stayed at the globular stage even when wild-type embryos develop to form embryonic cotyledons (Fig. 5m-n). These results suggested that CGF1 and CGF2 play roles in ovule development and embryogenesis to mediate fertility.

### *CGF1* and *CGF2* mediate leaf development

**Fig. 6:**
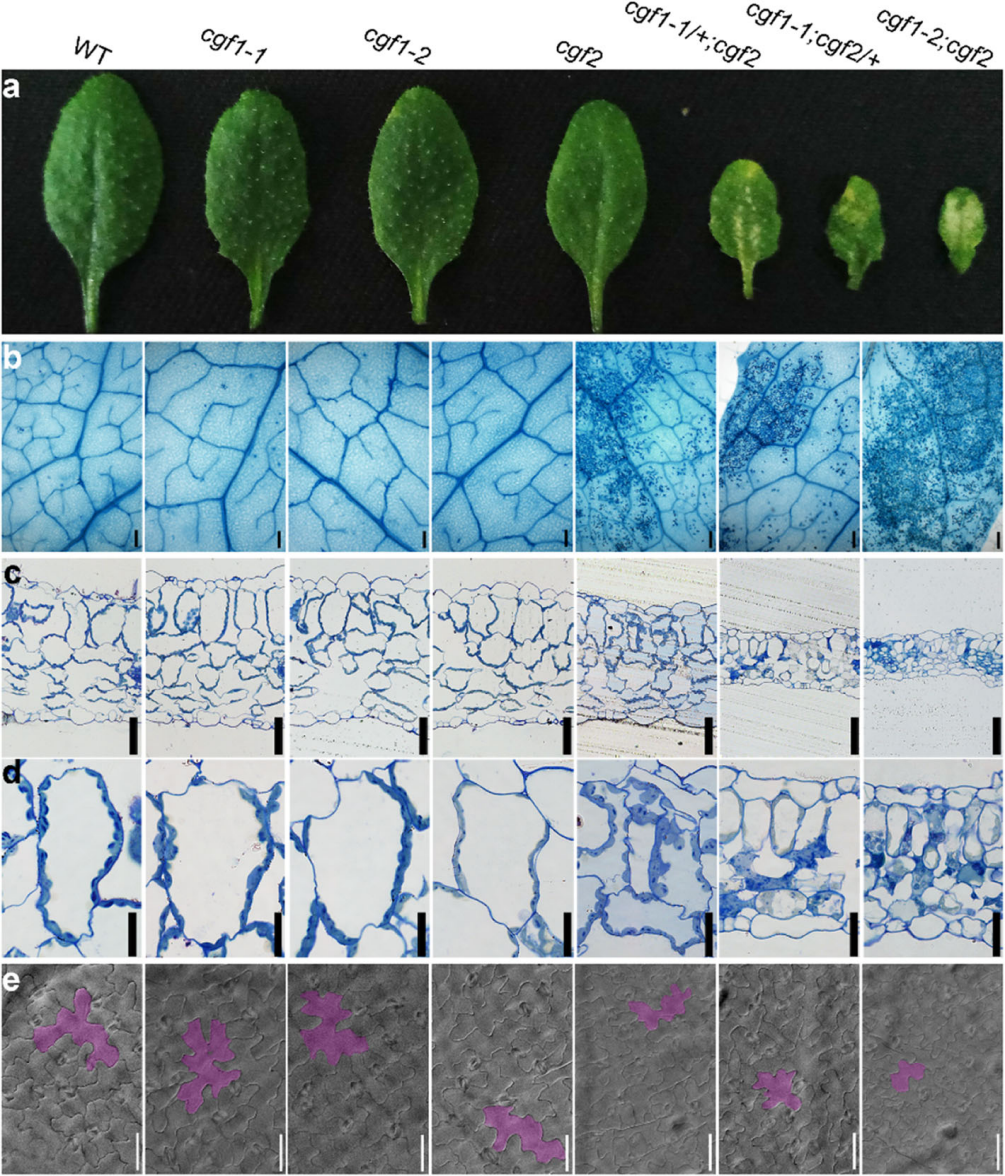
Mutations of both *CGF1* and *CGF2* affected leaf development. **a-e** A leaf **a**, trypan blue staining **b**, transverse semi-thin section **c-d**, or DIC **e** of the 4th true leaf from 3 WAG wild-type, *cgf1–1*, *cgf1–2*, *cgf2*, *cfg1–1*/+;*cgf2*, *cgf1–1*;*cgf2*/+, or *cgf1–2;cgf2* plants. **d** Mesophyll cells or epidermal pavement cells from 3 WAG wild-type, *cgf1–1*, *cgf1–2*, *cgf2*, *cfg1–1*/+;*cgf2*, *cgf1–1*;*cgf2*/+, or *cgf1–2;cgf2* plants. One pavement cell is artificially colored to highlight in **e**. Bars = 200 μm for **b**; 50 μm for **c, e**; 20 μm for **d**

Both the homozygous *cgf1–2;cgf2* and the haploinsufficient mutants *cgf1–1*/+;*cgf2* and *cgf1–1*;*cgf2*/+ are compromised in leaf morphology (Fig. 4). To gain a better understanding of the physiological role of *CGF1* and *CGF2*, we analyzed leaf development in details. Leaves of the three double mutants were smaller (Figure S6). Large yellow patches appeared on the leaves of the three double mutants but not on those of single mutants (Fig. 6a). Trypan blue staining indicated that these yellow patches were areas of cell death (Fig. 6b). Cross-section and transmission electron micrographs (TEM) of leaves showed a significant reduction in leaf thickness and palisade cell size (Fig. 6c, Figure S6). A substantial portion of mesophyll cells, especially the palisade and spongy layers, showed cell death in the three double mutants but not in wild type or single mutants (Fig. 6d). Observation with differential interference contrast (DIC) microscopy on cleared leaves showed that pavement cell size was significantly reduced in the three double mutants in comparison to that of wild type or single mutants (Fig. 6e, Figure S6). These results demonstrated a key role of CGF1 and CGF2 in leaf development.

### *CGF1* and *CGF2* mediate chloroplast integrity

**Fig. 7:**
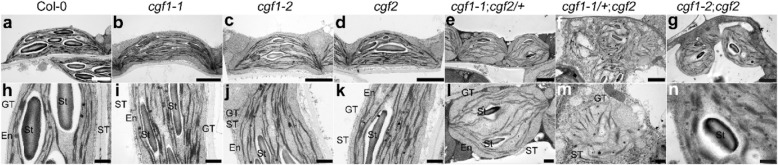
Mutations of both *CGF1* and *CGF2* compromised chloroplast integrity. **a-n** Representative TEM of chloroplasts from wild-type, *cgf1–1*, *cgf1–2*, *cgf2*, *cgf1–1*;*cgf2*/+, *cfg1–1*/+;*cgf2*, or *cgf1–2;cgf2* plants. Bars = 2 μm for **a-g**; 500 nm for **h-n**. En, envelop; GT, grana thylakoids; ST, stroma thylakoids; St, starch

Because CGF1 and CGF2 are targeted to the chloroplasts (Fig. 3, Figure S3) and mutations caused yellow patches on leaves (Fig. 6), we therefore wondered whether chloroplasts were affected by mutations of *CGF*s. To this purpose, we performed TEMs on maturing leaves of 3 weeks after germination (WAG) plants. Compared to wild type, the three double mutants contained a significantly reduced chloroplast number (Figure S7). Chloroplasts in wild-type cells possessed integral envelops and well-developed thylakoid membranes with grana connected by stroma lamellae (Fig. 7a, h), as did the single mutants, i.e. *cgf1–1* (Fig. 7b, i), *cgf1–2* (Fig. 7c, j), and *cgf2* (Fig. 7d, k). By contrast, chloroplasts in *cgf1–1;cgf2*/+ (Fig. 7e, l), *cgf1–1*/+;*cgf2* (Fig. 7f, m), and *cgf1–2;cgf2* (Fig. 7g, n) showed defects to various degree. Morphology of chloroplasts changed from spindles to spheres (Fig. 7l, m, n). Membrane structure of chloroplasts and thylakoid membranes were abnormal (Fig. 7 l, m, n). The percentage of damaged chloroplasts was significantly high in the three mutants compared to either wild type or single mutants (Figure S7). These results suggested that *CGF1* and *CGF2* are critical for the maintenance of chloroplast integrity.

**Fig. 8:**
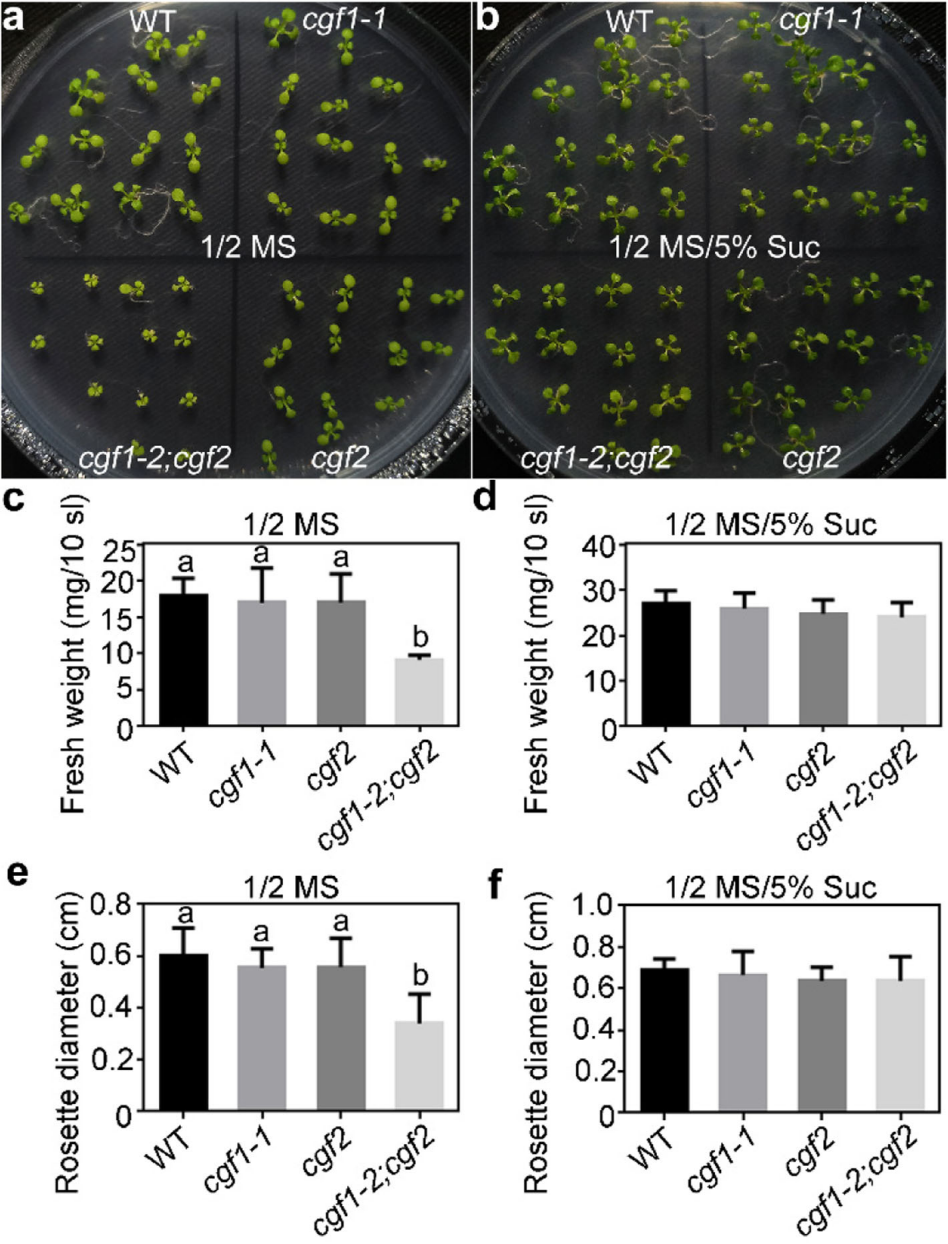
Exogenous sucrose partially rescued the reduced growth of *cgf1–2*;*cgf2*. **a-b** Representative seedling growth on 1/2 MS medium **a** or on 1/2 MS supplemented with 5% sucrose **b**. **c-d** Fresh weight at 10 DAG of ten wild type, *cfg1–1*, *cgf2*, or *cgf1–2;cgf2* seedlings. **e-f** Rosette diameter at 10 DAG of wild type, *cfg1–1*, *cgf2*, or *cgf1–2;cgf2* seedlings. Results are means ± SE (*n* > 6). Different letters indicate significant different groups (OneWay ANOVA, Tukey’s multiple comparisons test, *P* < 0.05)

Because of the compromised chloroplast integrity, the contents of chlorophyll and starch were also significantly decreased (Figure S7). To determine whether defective growth of the *cgf1–2*;*cgf2* double mutant was due to limited carbon supply as indicated by the reduced chlorophyll and starch (Figure S7), we applied exogenous sucrose to the growth medium. Indeed, a higher sucrose could restore the growth of *cgf1–2;cgf2* such that its fresh weight and rosette diameter were comparable to wild type (Fig. 8), suggesting that defective vegetative growth by mutations of *CGFs* was resulted from reduced carbon supply due to chloroplast defects.

## Discussion

In this study, we reported the characterization of two nuclear-encoded chloroplast proteins, which are critical for the development and fertility of Arabidopsis. Mutations of Arabidopsis *CGF1* and *CGF2* most prominently affected leaf development (Fig. 4; Figure S4). In addition to smaller leaves from smaller cells, substantial cell death was detected when *CGF1* and *CGF2* were mutated. This was indicated by yellow patches on leaves, trypan blue staining of leaves, as well as disintegration of mesophyll cells from transverse sections of leaves (Fig. 6; Figure S6). Such defects are likely due to limited carbon supply of the double mutant. Indeed, exogenous sucrose restored seedling growth of the *cgf1–2*;*cgf2* double mutants (Fig. 8), confirming the hypothesis. By using TEMs, we further demonstrated that mutations of Arabidopsis *CGF1* and *CGF2* affected chloroplast integrity (Fig. 7; Figure S7), consistent with them being chloroplast integral proteins (Fig. 3; Figure S3). Only the double mutants of *CGF1* and *CGF2* showed growth defects (Fig. 4), suggesting their functional redundancy. Interestingly, the expression of *CGF1* was significantly increased in the *cgf2* mutant (Figure S4), suggesting a compensation program for these two functionally redundant genes.

Both *CGF1* and *CGF2* are expressed in non-greening tissues and cells, such as ovules and developing seeds (Fig. 2, Figure S2), where proteins they encode reside in plastids (Fig. 3, Figure S3). These results indicated the roles of CGF1 and CGF2 in other developmental processes. Indeed, the absence of the *cgf1–1*;*cgf2* double mutant (Table S1) indicates seedling lethality by *CGF* loss-of-function. Seed germination and greening involve the development of chloroplasts within 30 min after exposure to light [20] and failure of chloroplast development often leads to seedling lethality [21]. It is likely that CGFs are critical for the proplastid-chloroplast conversion during seed germination and greening. An additional line of evidence is that chloroplasts from mature leaves of the double *cgf* mutants were roundish without clear starch granules (Fig. 7), similar to those from newly initiated wild-type leaves [22], suggesting the involvement of *CGF1* and *CGF2* in chloroplast development.

We also report an unexpected role of chloroplast-associated proteins in female gametogenesis, i.e. embryo sac development. A significantly higher number of ovules from *cgf1–1*/+;*cgf2*, *cgf1–1*;*cgf2*/+, and *cgf1–1*;*cgf2* contained defective embryo sacs, which led to reduced female fertility (Fig. 5). We consider it likely that a limited carbon supply may have caused such defects, similar to what have been observed during seedling growth (Fig. 8). The limited carbon supply would be local rather than from vegetative tissues because female fertility is less affected in *cgf1–2*;*cgf2* than in the two heterozygous mutants *cgf1–1*/+;*cgf2* and *cgf1–1*;*cgf2*/+ (Fig. 5), both of which showed a less affected vegetative growth defect than *cgf1–2*;*cgf2* (Fig. 4). An alternative possibility is that CGF1/2 may participate in retro-signaling from chloroplasts to nuclear gene expression, a scenario worthy of future investigation.

## Conclusion

This study reports that two nuclear-encoded chloroplast proteins, Chloroplast protein for Growth and Fertility (CGF1) and CGF2, play important roles in vegetative growth, in female gametogenesis, and in embryogenesis likely by mediating chloroplast integrity and development.

## Method

### Plant growth and transformation

*Arabidopsis thaliana* ecotype Columbia-0 (Col-0), obtained from Arabidopsis Biological Resource Center (ABRC, www.arabidopsis.org), was used as wild type for all experiments in this study. Mutants including *cgf1–1*, *cgf1–2*, and *cgf2* were generated from Col-0 by CRISPR/Cas9. Genotyping of all mutants was performed by sequencing with appropriate primers (Supplemental Table 2). Transgenic plants were selected on half-strength Murashige and Skoog medium (1/2 MS) supplemented with either 25 μg/mL hygromycin B or 30 μg/mL Basta salt (Sigma-Aldrich). Surface-sterilized Arabidopsis seeds were planted on 1/2 MS containing 1% (w/v) sucrose and 1% (w/v) agar (pH 5.8). After stratifying at 4 °C for 2 days, the plants were transferred to a growth chamber. For soil growth, seedlings at 7 DAG on 1/2 MS were transferred to nutrient-rich soil in greenhouse with normal light conditions (90 μmol/m^2^/s) at a long-day cycle (16 h light/8 h dark) at 22 °C.

### DNA manipulation

All constructs were generated using the Gateway technology (Invitrogen) except for the CRISPR/Cas9 constructs. The pENTR/D/TOPO vector (Invitrogen) was used to generate all entry vectors. For the genomic-GUS constructs, the entry vector for *CGF1g* or *CGF2g* contains 3041 bp or a 1985 bp sequence of the *CGF1* or *CGF2* genomic locus (the primer pair ZP5837/ZP4998 for *CGF1g* and ZP5838/ZP5739 for *CGF2g*), which includes a 1415 bp or 622 bp sequence upstream of the corresponding translational start codon. For the *CGF2g* construct used for complementation, a 2193 bp genomic fragment of *CGF2* containing 3′-UTR was amplified with the primer pair ZP5838/ZP9022. Entry vectors were used in LR reactions with the destination vector pMD163 [23] to generate *CGF1g*-GUS, *CGF2g*-GUS, or *CGF2g* respectively.

For constructs used in subcellular localization, the coding sequences of *CGF1*, *CGF2*, and various mutant forms were amplified with the following primer pairs: ZP4997/ZP4998 for *CGF1*, ZP5738/ZP5739 for *CGF2*, ZP4997/ZP9786 for *CGF1*^*SP*^, ZP5738/ZP9784 for *CGF2*^*SP*^, ZP9787/ZP4998 for *CGF1*^*ΔSP*^, and ZP9785/ZP5739 for *CGF2*^*ΔSP*^. The resultant entry vectors were used in LR reactions with the destination vector *Pro*_*UBQ10*_:GW-GFP [24] or *Pro*_*35S*_:GW-GFP [25] to generate expression vectors, including *Pro*_*UBQ10*_:CGF1-GFP, *Pro*_*UBQ10*_:CGF2-GFP, *Pro*_*35S*_:CGF1^SP^-GFP, *Pro*_*35S*_:CGF2^SP^-GFP, *Pro*_*35S*_:CGF1^ΔSP^-GFP, and *Pro*_*35S*_:CGF2^ΔSP^-GFP.

The CRISPR constructs used to generate mutants of *CGF1* or *CGF2* were as described [26]. Briefly, the two target sites, one for *CGF1* and the other for *CGF2*, were selected using an online bioinformatics tool (http://www.genome.arizona.edu/crispr/CRISPRsearch.html) and were incorporated into forward and reverse PCR primers. The CGF1/CGF2-CRISPR cassette was generated by PCR amplifications from pCBC-DT1T2 with the primer pairs ZP5839/ZP5840 and ZP5841/ZP5842. The PCR products were digested with *Bsa*I and inserted into pHSE401, resulting in pHSE401-CGF1/CGF2. All entry vectors were sequenced. All primers are listed in Table S2.

### qPCRs

For qPCRs of *CGF1* and *CGF2* at different tissues, total RNAs were isolated from seedlings and roots at 7 DAG, leaves at 14 DAG, stems at 25 DAG, and reproductive tissues at 4–5 days after anthesis. For qPCRs analyzing the expression of *CGF2* in *Pro*_*35S*_*:CGF1-RNAi*;*cgf2* plants, total RNAs were isolated from leaves at 14 DAG. Total RNAs were isolated using a Qiagen RNeasy plant mini kit according to manufacturer’s instructions. Oligo (dT)-primed cDNAs were synthesized using Superscript III reverse transcriptase with on-column DNase II digestion (Invitrogen). The qRT-PCRs were performed with the Bio-Rad CFX96 real-time system using SYBR Green real-time PCR master mix (Toyobo) as described [27]. The specific primers used for *CGF1* and *CGF2* are ZP9333/ZP9334 and ZP5013/ZP5014, respectively. *GAPDH* and *TUBLIN2* were used as internal controls. All experiments were repeated in three biological replicates with similar results. All primers are listed in Table S2.

### Histochemical GUS staining

For the histochemical GUS analysis, different tissues (seedlings at 7 DAG, leaves at 14 DAG, inflorescence, and pistils) of the CGF1g-GUS and CGF2g-GUS transgenic plants were performed as described [27].

### Measurement and quantification

Fresh weights of 4 WAG plants were measured using an electronic microbalance. For the quantification of rosette diameter and rosette area, plants were photographed and measured with ImageJ (http://rsbweb.nih.gov/ij/). Imaging of leaf pavement pattern was performed as followed: five 4th true leaves from 3 WAG plants were fixed in 15% acetic acid:85% ethanol for overnight; washed in 70% ethanol and sequentially in absolute ethanol; cleared in Chloral Hydrate solution (200 g chloral hydrate, 20 g glycerin, and 50 g H_2_O) for one week; washed twice in 70% ethanol; mounted on slides in 50% glycerol; visualized with a Zeiss Axiophot microscope. Quantification of palisade cell diameter, palisade cell density, and epidermal cell size was measured using ImageJ. Leaf thickness was measured from transverse sections of leaves from 3 WAG plants using ImageJ. The number of chloroplasts per cell and percentage of damaged chloroplasts were measured from TEMs of 3 WAG leaves using ImageJ. For the measurement of chlorophyll contents, 2 g rosette leaves were harvested from 3 WAG plants. Chlorophyll contents were measured using the spectrophotometry as described [28]. For the measurement of starch contents, rosette leaves were harvested from 3 WAG plants. Starch contents was measured using anthrone colorimetry.

### Phenotype analysis

Pollen development by Alexander staining, 4′,6-diamino-phenylindole (DAPI) staining, SEM were performed as described previously [29]. Whole-mount embryo clearing were performed as described [19]. CLSM of optical sections were performed as described [18]. The 4th true leaves of 3 WAG plants were cut into small pieces for plastic sections and TEMs as performed as described [27, 30].

### Imaging

CLSM were captured using a Zeiss LSM880 laser scanning microscope with a 40/1.3 oil objective. Fluorescence of GFP and auto-fluorescence of chloroplast were captured using the excitation/emission settings: 488 nm/505–550 nm for GFP, 561 nm/600–650 nm for chloroplast. Differential interference contrast (DIC) imaging of leaves were performed using a Zeiss Axiophot microscope with DIC optics.

### Phylogenetic analysis

Phylogenetic analysis was performed using MEGA7.0 based on protein sequences of CGF homologs.

### Accession number

Arabidopsis Genome Initiative locus identifiers for the genes mentioned in this article are: At4g35080 for *CGF1*; At2g16800 for *CGF2*.

## Supplementary information

**Additional file 1 Figure S1.** CGF1 and CGF2 are homologous with multiple transmembrane domains predicted. **Figure S2.***CGF1* and *CGF2* are expressed in diverse tissues and developmental stages. **Figure S3.** CGF2 targets to chloroplasts through its N-terminal sequences. **Figure S4.** Downregulating *CGF1* in *cgf2* mimicked defects of the double mutants. **Figure S5.** Mutations of *CGF1* and *CGF2* did not affect pollen development. **Figure S6.** Mutations of both *CGF1* and *CGF2* affected leaf development. **Figure S7.** Mutations of both *CGF1* and *CGF2* compromised chloroplast integrity. **Table S1.** Segregation ratio. **Table S2.** Oligos used in this study.

## Data Availability

The datasets used and/or analysed during the current study available from the corresponding author on reasonable request.

## Abbreviations

CGF: Chloroplast protein for growth and fertility
CLSM: Confocal laser scanning micrograph
CRISPR: Clustered regularly interspaced short palindromic repeats
cTP: Chloroplast transit peptide sequence
DAG: Days after germination
DIC: Differential interference contrast
GFP: Green fluorescence protein
SP: Signal peptide
TEM: Transmission electron micrographs
TM: Transmembrane

## References

[1] Block MA, Douce R, Joyard J, Rolland N (2007). Chloroplast envelope membranes: a dynamic interface between plastids and the cytosol. Photosyn Res.

[2] Hölzl G, Dörmann P (2019). Chloroplast lipids and their biosynthesis. Annu Rev Plant Biol.

[3] Bobik K, Burch-Smith TM. Chloroplast signaling within, between and beyond cells. Front Plant Sci. 2015;6:781. 10.3389/fpls.2015.00781 PMC4593955 26500659

[4] Li Q, Yao ZJ, Mi H (2016). Alleviation of photoinhibition by co-ordination of chlororespiration and cyclic electron flow mediated by NDH under heat stressed condition in tobacco. Front Plant Sci.

[5] Wang W, Tang W, Ma T, Niu D, Jin JB, Wang H, Lin R (2016). A pair of light signaling factors FHY3 and FAR1 regulates plant immunity by modulating chlorophyll biosynthesis. J Integr Plant Biol.

[6] Wang Z, Wang F, Hong Y, Huang J, Shi H, Zhu J-K (2016). Two chloroplast proteins suppress drought resistance by affecting ROS production in guard cells. Plant Physiol.

[7] Ball S, Colleoni C, Cenci U, Raj JN, Tirtiaux C (2011). The evolution of glycogen and starch metabolism in eukaryotes gives molecular clues to understand the establishment of plastid endosymbiosis. J Exp Bot.

[8] Ball SG, Subtil A, Bhattacharya D, Moustafa A, Weber APM, Gehre L, Colleoni C, Arias M-C, Cenci U, Dauvillée D (2013). Metabolic effectors secreted by bacterial pathogens: essential facilitators of plastid endosymbiosis?. Plant Cell.

[9] Nakayama T, Archibald JM (2012). Evolving a photosynthetic organelle. BMC Biol.

[10] Yoon HS, Hackett JD, Ciniglia C, Pinto G, Bhattacharya D (2004). A molecular timeline for the origin of photosynthetic eukaryotes. Mol Biol Evol.

[11] Wu W, Elsheery N, Wei Q, Zhang L, Huang J (2011). Defective etioplasts observed in variegation mutants may reveal the light-independent regulation of white/yellow sectors of Arabidopsis leaves. J Integr Plant Biol.

[12] Zhang L, Kato Y, Otters S, Vothknecht UC, Sakamoto W (2012). Essential role of VIPP1 in chloroplast envelope maintenance in Arabidopsis. Plant Cell.

[13] Trösch R, Jarvis P (2011). The stromal processing peptidase of chloroplasts is essential in Arabidopsis, with knockout mutations causing embryo arrest after the 16-cell stage. PLoS One.

[14] Qi Y, Wang X, Lei P, Li H, Yan L, Zhao J, Meng J, Shao J, An L, Yu F (2020). The chloroplast metalloproteases VAR2 and EGY1 act synergistically to regulate chloroplast development in Arabidopsis. J Biol Chem.

[15] Lee K, Park SJ, Han JH, Jeon Y, Pai HS, Kang H (2019). A chloroplast-targeted pentatricopeptide repeat protein PPR287 is crucial for chloroplast function and Arabidopsis development. BMC Plant Biol.

[16] Kleffmann T, Russenberger D, von Zychlinski A, Christopher W, Sjolander K, Gruissem W, Baginsky S (2004). The *Arabidopsis thaliana* chloroplast proteome reveals pathway abundance and novel protein functions. Curr Biol : CB.

[17] Liu H-H, Xiong F, Duan C-Y, Wu Y-N, Zhang Y, Li S (2019). Importin β4 mediates nuclear import of GRF-interacting factors to control ovule development in Arabidopsis. Plant Physiol.

[18] Wang J-G, Feng C, Liu H-H, Ge F-R, Li S, Li H-J, Zhang Y (2016). HAPLESS13-mediated trafficking of STRUBBELIG is critical for ovule development in Arabidopsis. PLoS Genet.

[19] Xiong F, Liu H-H, Duan C-Y, Zhang B-K, Wei G, Zhang Y, Li S (2019). Arabidopsis JANUS regulates embryonic pattern formation through Pol II-mediated transcription of *WOX2* and *PIN7*. iScience.

[20] Reinbothe S, Reinbothe C, Lebedev N, Apel K (1996). PORA and PORB, two light-dependent protochlorophyllide-reducing enzymes of angiosperm chlorophyll biosynthesis. Plant Cell.

[21] Kwon KC, Cho MH (2008). Deletion of the chloroplast-localized AtTerC gene product in *Arabidopsis thaliana* leads to loss of the thylakoid membrane and to seedling lethality. Plant J.

[22] Pogson BJ, Ganguly D, Albrecht-Borth V (2015). Insights into chloroplast biogenesis and development. Biochim Biophys Acta.

[23] Curtis MD, Grossniklaus U (2003). A gateway cloning vector set for high-throughput functional analysis of genes *in planta*. Plant Physiol.

[24] Feng Q-N, Song S-J, Yu S-X, Wang J-G, Li S, Zhang Y (2017). Adaptor protein-3-dependent vacuolar trafficking involves a subpopulation of COPII and HOPS tethering proteins. Plant Physiol.

[25] Karimi M, Inzé D, Depicker A (2002). GATEWAY™ vectors for *Agrobacterium*-mediated plant transformation. Trends Plant Sci.

[26] Xing HL, Dong L, Wang ZP, Zhang HY, Han CY, Liu B, Wang XC, Chen QJ (2014). A CRISPR/Cas9 toolkit for multiplex genome editing in plants. BMC Plant Biol.

[27] Zhou L-Z, Li S, Feng Q-N, Zhang Y-L, Zhao X, Zeng Y-L, Wang H, Jiang L, Zhang Y (2013). PROTEIN S-ACYL TRANSFERASE10 is critical for development and salt tolerance in *Arabidopsis*. Plant Cell.

[28] Zhao X-Y, Wang J-G, Song S-J, Wang Q, Kang H, Zhang Y, Li S (2016). Precocious leaf senescence by functional loss of *PROTEIN S-ACYL TRANSFERASE14* involves the NPR1-dependent salicylic acid signaling. Sci Rep.

[29] Li S, Ge FR, Xu M, Zhao XY, Huang GQ, Zhou LZ, Wang JG, Kombrink A, McCormick S, Zhang XS (2013). Arabidopsis COBRA-LIKE 10, a GPI-anchored protein, mediates directional growth of pollen tubes. Plant J.

[30] Wang J-G, Li S, Zhao X-Y, Zhou L-Z, Huang G-Q, Feng C, Zhang Y (2013). HAPLESS13, the Arabidopsis μ1 adaptin, is essential for protein sorting at the *trans*-Golgi network/early endosome. Plant Physiol.

