# Supplementary material for: Arabidopsis Chloroplast protein for Growth and Fertility1 (CGF1) and CGF2 are essential for chloroplast development and female gametogenesis
Source: BMC Plant Biol. 2020 Apr 19;20:172. doi: 10.1186/s12870-020-02393-5 (PMC7168881; doi:10.1186/s12870-020-02393-5)
Supplement: Supplementary file 1 — Additional file 1 Figure S1. CGF1 and CGF2 are homologous with multiple transmembrane domains predicted. Figure S2.CGF1 and CGF2 are expressed in diverse tissues and developmental stages. Figure S3. CGF2 targets to chloroplasts through its N-terminal sequences. Figure S4. Downregulating CGF1 in cgf2 mimicked defects of the double mutants. Figure S5. Mutations of CGF1 and CGF2 did not affect pollen development. Figure S6. Mutations of both CGF1 and CGF2 affected leaf development. Figure S7. Mutations of both CGF1 and CGF2 compromised chloroplast integrity. Table S1. Segregation ratio. Table S2. Oligos used in this study. [file 12870_2020_2393_MOESM1_ESM.pdf]

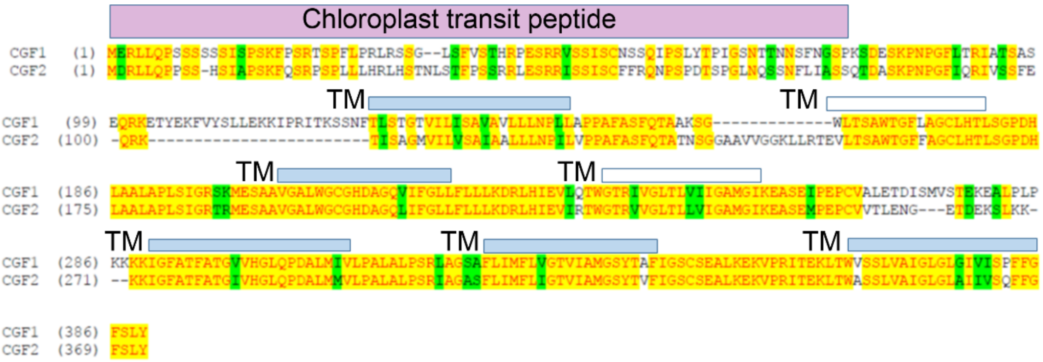

Supplemental Figure 1. CGF1 and CGF2 are homologous with multiple transmembrane domains predicted.

Sequence alignment was performed with VectorNTI. Yellow-highlighted amino acids are identical while green-highlighted amino acids are similar in side chains. Lilac box indicates predicted chloroplast transit peptide (TargetP). Blue boxes indicate TM domains shared by both proteins while empty boxes indicate TM domains only predicted in CGF1 (HMMTOP).

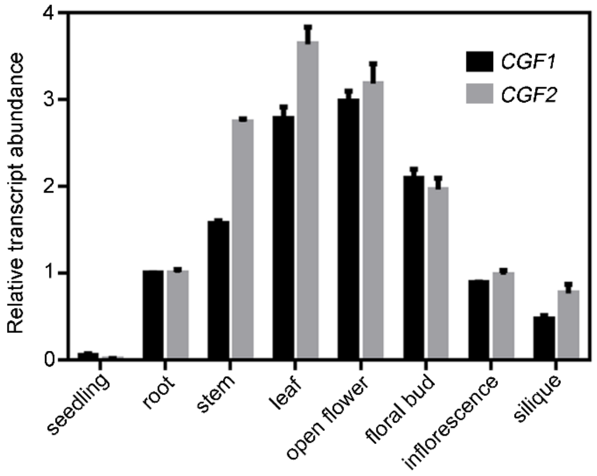

Supplemental Figure 2. *CGF1* and *CGF2* are expressed in diverse tissues and developmental stages.

Quantitative real-time PCRs (qPCRs) of *CGF1* and *CGF2* among different tissues. Results shown are means ± standard errors (SEM, n=3). Each biological replicates were repeated three times with similar results.

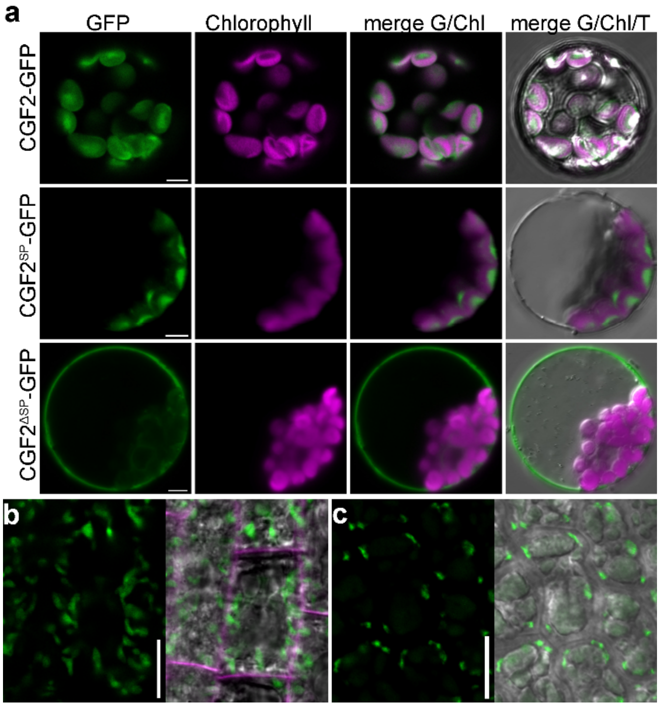

Supplemental Figure 3. CGF2 targets to chloroplasts through its N-terminal sequences. (a) CLSM of protoplasts from *Pro*$_{UBQ10}$:CGF2-GFP, *Pro*$_{35S}$:CGF2^{SP}-GFP*, or Pro*$_{35S}$:CGF2$^{\Delta SP}$-GFP transgenic plants. From left to right: the GFP channel, autofluorescence channel (chlorophyll), merge of the GFP and autofluorescence (Chl) channels, merge of the GFP, autofluorescence, and transmission channels. (b) CLSM of root epidermal cells from *Pro*$_{UBQ10}$:CGF2-GFP. The right image is the merge of the GFP, RFP (FM4-64), and transmission channels. (c) CLSM of pavement cells from embryonic cotyledons of the *Pro*$_{UBQ10}$:CGF2-GFP transgenic plants. The right image is the merge of the GFP and transmission channels. Bars = 5 µm for (a); 10 µm for (b-c).

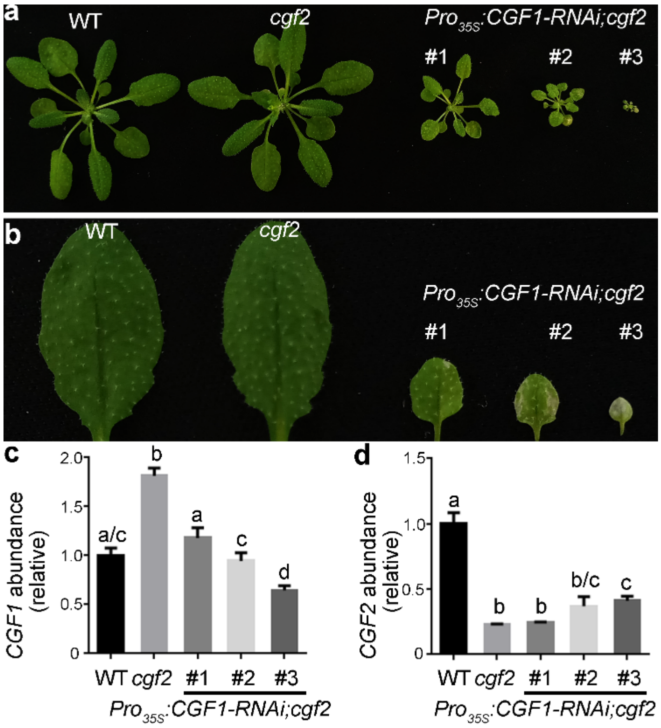

Supplemental Figure 4. Downregulating *CGF1* in *cgf2* mimicked defects of the double mutants.

(a-b) A representative 4 WAG plant (a) or the 6th true leaf (b) from 4 WAG wild-type, *cgf2*, or three lines of the *Pro$_{35S}$*:CGF1-RNAi;*cgf2* transgenic plants. (c-d) Relative *CGF1* (c) or *CGF2* (d) transcript abundance by qPCRs. Results are means ± SEM (n=3). Each biological replicates were repeated three times with similar results. Different letters indicate significant different groups (OneWay ANOVA, Tukey's multiple comparisons test, $P<0.05$).

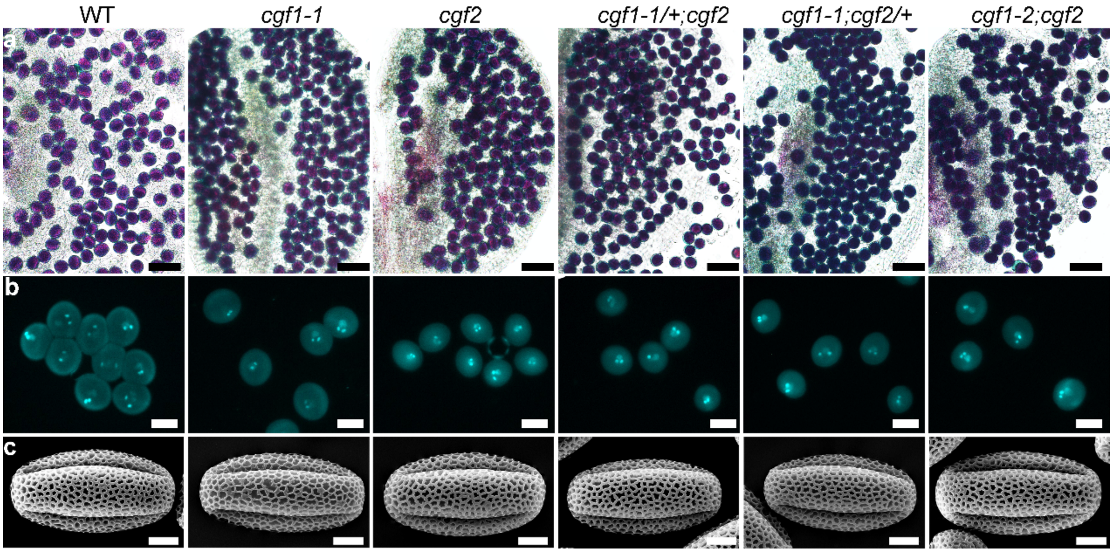

Supplemental Figure 5. Mutations of *CGF1* and *CGF2* did not affect pollen development.

(a-c) Alexander staining of a dehiscing anther (a), DAPI staining of mature pollen grains (b), or scanning electron micrographs (SEM, c) from wild-type, *cgf1-1*, *cgf1-2*, *cgf2*, *cfg1-1*/+;*cgf2*, *cgf1-1*;*cgf2*/+, or *cgf1-2;cgf2* plants. Bars = 50 µm for (a); 20 µm for (b); 5 µm for (c).

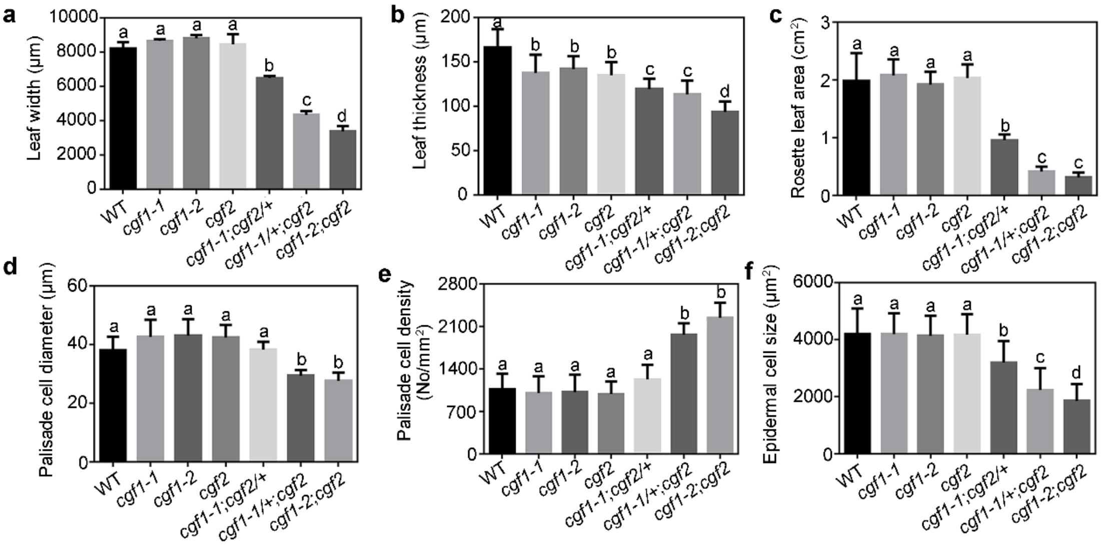

Supplemental Figure 6. Mutations of both *CGF1* and *CGF2* affected leaf development. (a-f) Quantification of leaf width (a), leaf thickness (b), rosette area (c), palisade cell diameter (d), palisade cell density (e), or pavement cell size (f) of the 4th true leaf from 3 WAG wild-type, *cgf1-1*, *cgf1-2*, *cgf2*, *cfg1-1*/+;*cgf2*, *cgf1-1*;*cgf2*/+, or *cgf1-2;cgf2* plants. Results are means ± SE for (a) (n >4); for (d) and (e) (n > 3). Results are means ± SD for (b) (n > 75); for (c) (n > 8); for (f) (n > 65). Different letters indicate significant different groups (OneWay ANOVA, Tukey's multiple comparisons test, P<0.05).

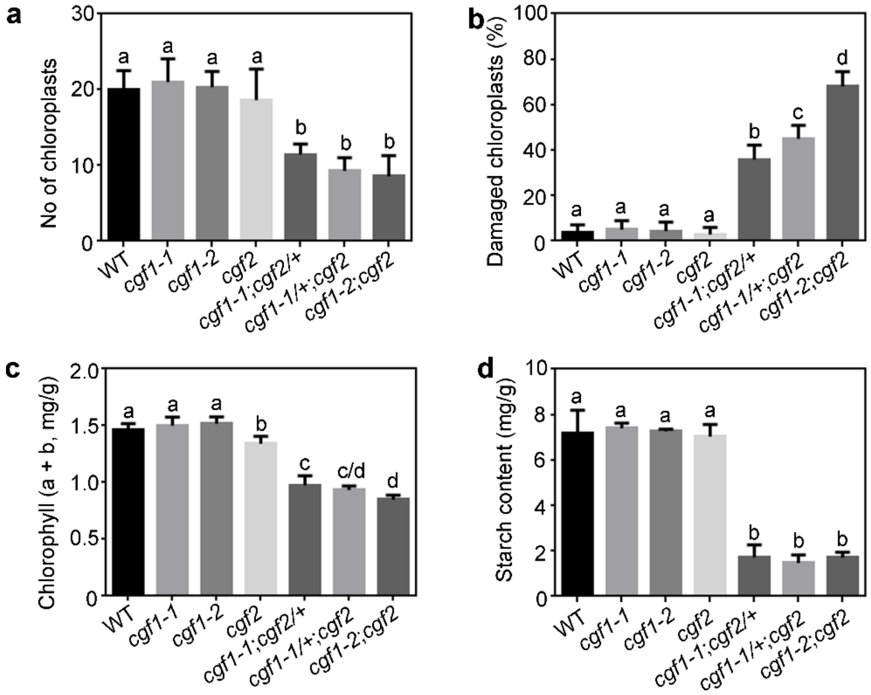

Supplemental Figure 7. Mutations of both *CGF1* and *CGF2* compromised chloroplast integrity.

(a-d) Number of chloroplasts per cross-section (a), the percentage of damaged chloroplasts among total chloroplasts (b), chlorophyll content (c), or starch content (d) of the 4th true leaf from 3 WAG wild-type, *cgf1-1*, *cgf1-2*, *cgf2*, *cfg1-1*/+;*cgf2*, *cgf1-1*;*cgf2*/+, or *cgf1-2;cgf2* plants. Results are means ± SE (n > 7). Different letters indicate significant different groups (OneWay ANOVA, Tukey's multiple comparisons test, P<0.05).

Supplemental Table 1. Segregation ratio.

Parents	Progenies		
Female X Male	Genotype	Expected ratio	Observed
AaBb X AaBb	A(A/a)B(B/b): aaB(B/b): A(B/b)bb: aabb	9: 3: 3: 1	397: 101: 125: 0[a]
Aabb X Aabb	AAbb: Aabb: aabb	1: 2: 1	68: 73: 0[b]
aaBb X aaBb	aaBB: aaBb: aabb	1: 2: 1	76: 89: 0[b]

[a] Significantly different from 9: 3: 3: 1 (χ^2, P< 0.01).

[b] Significantly different from 1: 2: 1 (χ^2, P< 0.01).

A: *CGF1*; B: *CGF2*; a: *cgf1-1*; b: *cgf2*.

Supplemental Table 2. Oligos used in this study.

**Application**		**No.**	**5'-3' sequences**
Cloning	*CGF1g*	ZP5837	CACCGTGGGACTCACTGGATTGC
		ZP4998	GTAGAGGCTAAAACCAAAGAAAGGG
	CGF2g	ZP5838	CACCACTCTCCACAACACCACCAATG
		ZP5739	ATACAGGCTGAATCCAAAGAAC
	CGF2g-3'UTR	ZP5838	CACCACTCTCCACAACACCACCAATG
		ZP9022	GAGCAAATCAACCCTTAAGAGAGGAAC
	CGF1	ZP4997	CACCATGGAAAGGCTTCTTCAACCATC
		ZP4998	GTAGAGGCTAAAACCAAAGAAAGGG
	$CGF1^{SP}$	ZP4997	CACCATGGAAAGGCTTCTTCAACCATC
		ZP9786	TTTGGGAGACCCATTGAAGGAATTG
	$CGF2^{SP}$	ZP5738	CACCATGGATAGGCTTTTGCAACCTC
		ZP9784	AGAAGCAATCAAGAAATTACTACTCTG
	$CGF1^{\Delta SP}$	ZP9787	CACCTCGGATGAATCGAAACCTAATCCC
		ZP4998	GTAGAGGCTAAAACCAAAGAAAGGG
	$CGF2^{\Delta SP}$	ZP9785	CACCTCTCAAACCGATGCGTCGAAAC
		ZP5739	ATACAGGCTGAATCCAAAGAAC
	CGF1&CGF2-CRISPR	ZP5839	ATATATGGTCTCGATTGAACTGCAGCTAAATCCGGTGTT
		ZP5840	TGAACTGCAGCTAAATCCGGTGTTTTAGAGCTAGAAATAGC
		ZP5841	AACCAGGACTGAAGTATTGACACAATCTCTTAGTCGACTCTAC
		ZP5842	ATTATTGGTCTCGAAACCAGGACTGAAGTATTGACACAA
qPCR	*CGF1*	ZP9333	CGTCACTGTATACGCCTATAGG
		ZP9334	AATCCGGGATTAGGTTTCGATT
	CGF2	ZP5013	GCTTCTTCTCAAACCGATGCG
		ZP5014	GAAAGCTAGCGAAAGCAGGC
	GAPDH	ZP687	TGAAATCAAAAAGCTATCAAGG
		ZP688	CATCATCCTCGGTGTATCCAA
